# Experimental Research on Seismic Behavior of Seismic-Damaged Double-Deck Viaduct Frame Pier Strengthened with CFRP and Enveloped Steel

**DOI:** 10.3390/ma15238668

**Published:** 2022-12-05

**Authors:** Chengxiang Xu, Yongang Wu, Xiaoqiang Liu, Xuhui Hu, Bingyang Zhou

**Affiliations:** 1 School of Urban Construction, Wuhan University of Science and Technology, Wuhan 430065, China; 2 Department of Architectural Engineering, Shandong Vocational College of Science and Technology, Weifang 261053, China; 3 China Construction Seventh Engineering Division Corp. Ltd., Zhengzhou 450004, China

**Keywords:** double-deck viaduct frame pier (DVFP), reinforcement, seismic damage, seismic behavior, low reversed cyclic loading

## Abstract

This paper investigates the seismic behavior of a seismic-damaged double-deck viaduct frame pier (DVFP) strengthened with CFRP and enveloped steel, four strengthened DVFP specimens with different degrees of initial damage were tested under quasi-static cyclic loading. Based on the test results, the hysteretic behavior, the stiffness and strength degradation, crack propagation, and failure mechanism were firstly analyzed. Then, the damage indexes of the tested specimens were calculated with different models to evaluate the seismic strengthening performance. Results of this study show that CFRP and enveloped steel strengthening could effectively improve the strength and ductility of pre-damaged DVFPs. The ultimate load, the failure displacement and the displacement ductility of the moderately damaged specimen after being strengthened were found to increase by 120.74%, 35% and 32.33%, respectively. For the severely damaged specimens with CFRP and enveloped steel strengthening, the figures were 105.36%, 25.98% and 31.41%, respectively. The research results can provide reference for the hybrid strengthening application of seismic-damaged DVFP.

## 1. Introduction

Double deck viaducts can fully improve the utilization of space and materials, and effectively achieve traffic diversion and capacity expansion, which is a functional and economical bridge type [1]. The bridge pier is a key element in the lateral force resistance system of the bridge structure, and the damage of bridge pier during an earthquake can cause a long-term disruption to the transportation system. Several studies have been conducted on the seismic performance of strengthened seismic-damaged bridge structures, which showed that seismic reinforcement is a cost-effective sustainable approach to strengthen damaged bridges in seismic zones [2,3,4,5]. RC jackets can improve the bending and shear capacity of the original column, and have been applied to repair and strengthen damaged RC columns, however, the application of RC jackets was limited because of the efficiency decrease as the column height increases [6].

Enveloped steel jackets also have good durability, are low cost, and are convenient for construction. There are also some experimental results showing that damaged columns strengthened with steel jackets achieved certain improvements in their strength and ductility capacity as compared with the original column [7,8,9,10], and it is also described in detail in the Code for the Design of Strengthening Concrete Structures (GB 50367-2013) and the Technical Regulations for Seismic Reinforcement of Buildings (JGJ116-2009) [11,12]. Carbon fiber-reinforced polymer (CFRP) jackets, which have light weight and high strength, are an effective strengthened method to confine the core concrete and preventing the steel bars from buckling in RC structures. A series of test results indicated that the CFRP-strengthened bridge columns achieved larger lateral capacity, flexural ductility and energy dissipation [13,14,15,16,17].

With the strong earthquakes frequently occurring around the world, the seismic performance of repaired damaged piers has attracted great attention in the civil engineering community. It is of great engineering significance to select an appropriate retrofitting or strengthening technique of damaged piers and evaluate their seismic performance. Wang et al. conducted a comparative experimental study on the seismic performance of pre-damaged RC frames reinforced with CFRP and angle steel and pre-damaged RC frames reinforced with CFRP [18], Lu et al. compared the seismic performance of RC columns reinforced with CFRP and enveloped steel and RC columns reinforced with enveloped steel [19], and Wang and Lu found that the composite strengthening technique using CFRP and enveloped steel has better seismic performance than single-material strengthening techniques.

As discussed above, the seismic strengthening techniques have been well developed around the word, and the double-deck and multi-story viaduct piers have been widely used in existing bridge structures. However, the research on double deck viaduct piers is rare, especially on strengthened seismic-damage DVFP. The stress characteristics of the pier structures are different from those of the building structure. Therefore, the experimental study of the seismic performance of strengthened damaged DVFP can provide support for the seismic strengthening technology standards for double-deck viaduct piers.

In this paper, CFRP and enveloped steel were used to strengthen the damaged DFVP and conducted the seismic performance test. Firstly, the DVFP specimens were pre-damaged by the proposed static test to simulate different degrees of seismic damage, and then strengthened with CFRP and enveloped steel. Finally, the reinforced specimens were subjected to low circumferential reciprocal loading damage tests. The seismic performance of DVFP strengthened with CFRP and enveloped steel under different degrees of seismic damage are investigated, and the feasibility and effectiveness of the strengthening techniques using CFRP and enveloped steel for seismic damage of DVFP are discussed.

## 2. Experimental Program

### 2.1. Test Specimens

**Figure 1:**
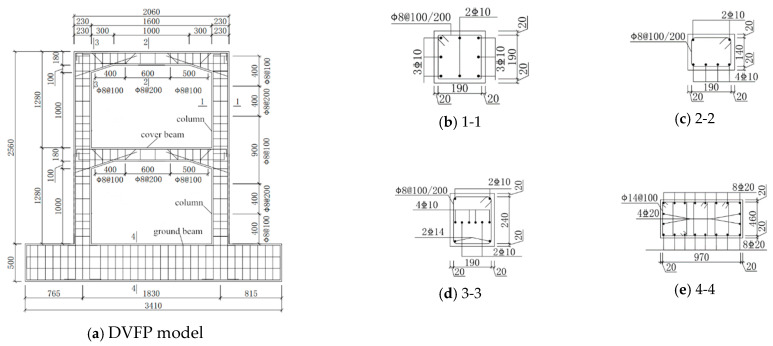
Details of DVFP specimen.

Four DVFP specimens with the same dimensions and reinforcement at 1:5.5 scale were designed and cast, and pre-damaged by static testing to simulate varying degrees of seismic damage, numbered from KJD-0 to KJD-3. The size and reinforcement condition of the specimens are shown in Figure 1. The longitudinal reinforcement ratio of column and cover beam are 1.19% and 1.14%, respectively, and the details of reinforcement are shown in Figure 1. The concrete strength of the specimen was C40 and the average tested compressive strength of the concrete cube at 28 days was 33.8 MPa (cube size: 150 mm × 150 mm × 150 mm). The mechanical properties of steel are shown in Table 1. The mechanical properties of CFRP, CFRP sheets mucilage and mucilage are shown in Table 2.

### 2.2. Test Device and Loading System

**Figure 2:**
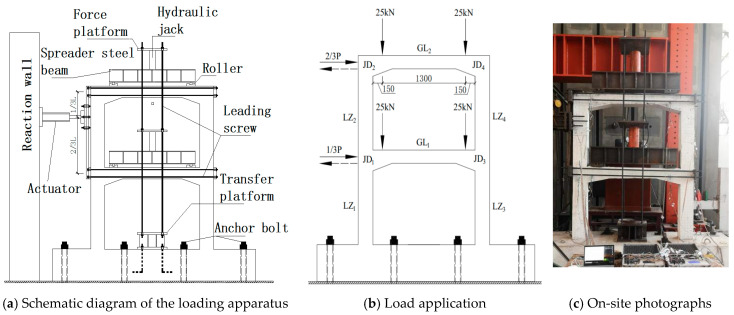
Loading device and scene.

The DVFP model was attached to the strong floor using 8 high-strength bolts. The vertical load adopted the self-reaction system, which is composed of oil jack, reaction platform, leading screw and spreader steel beam, the loading device. The test site is shown in Figure 2. The vertical loads were applied to the first and second floor cover beams, respectively. The horizontal cyclic load was applied by the actuator at the ends of the upper and lower cover beam in the proportion of 2:1 through the spreader device on the column side.

**Figure 3:**
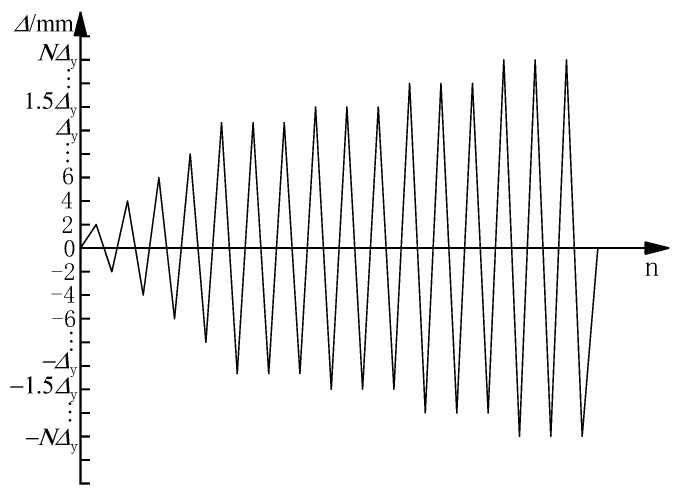
Loading history.

During the test, a constant axial load was applied at the cover beam on the first and second floors and the horizontal force was cycled under a lateral displacement control mode. Before the yield, one cycle was imposed with 2 mm and 1 time step for each loading step. After the specimen entered the yield stage, the lateral load repeated three cycles at the displacement levels of 0.5Δy, 1Δy, 1.5Δy, …, until the load decreased to less than 85% of the peak load or it failed. The loading system is shown in Figure 3.

### 2.3. Specimen Pre-Damage and Reinforcement

**Figure 4:**
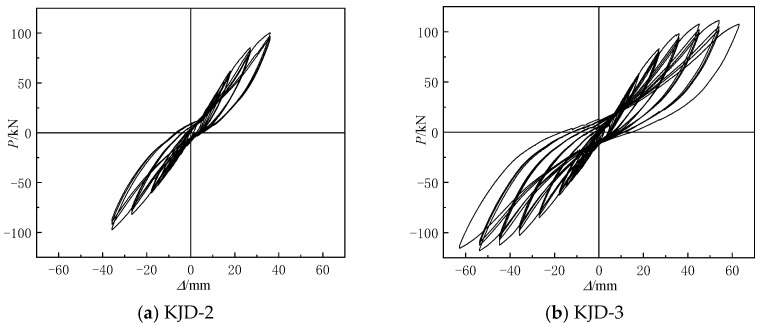
Hysteretic loops of pre-damage specimens.

The pre-damage parameters and reinforcement methods of the specimens are shown in Table 3. The specimen KJD-0 was not strengthened and specimen KJD-1 was reinforced directly without pre-damage. Specimens KJD-2 and KJD-3 were pre-damaged by quasi-static test to achieve a moderate damage and a severe damage of the reinforced concrete pier grade 5 performance class, respectively [20]. When the horizontal displacement reached 36 mm in the third cycle, cracks appeared at the column and joint of KJD-2, which was designated as moderate damage. When the specimen KJD-3 was loaded to ±63 mm in the first cycle, and the original cracks of joints and column foot extended, the concrete cover of the bottom column foot began to fall off, and the horizontal bearing capacity reached the peak load, which was designated as a severe damage. The hysteretic curves of pre-damage specimens are shown in Figure 4.

**Figure 5:**
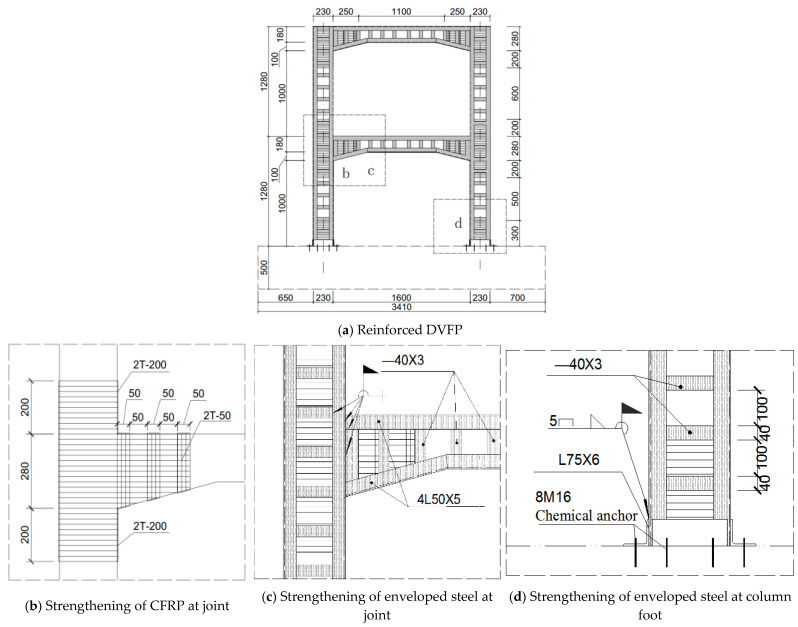
Reinforcement design of specimens.

**Figure 6:**
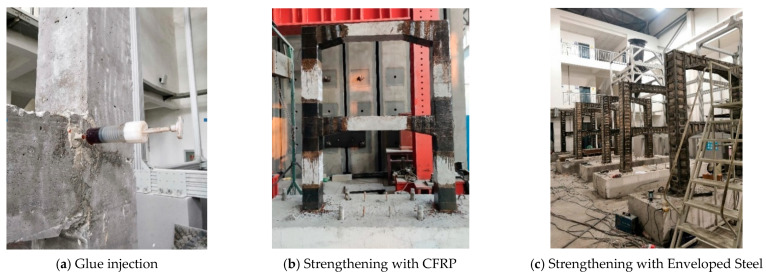

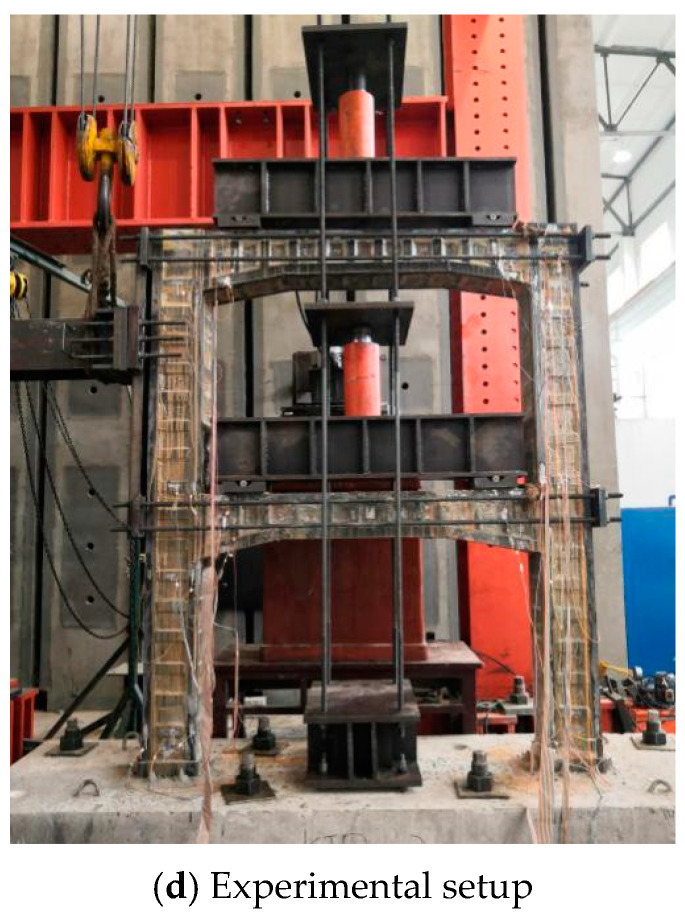
On-site photographs.

The reinforcement method of the specimens was based on references [20], specimens KJD-1, KJD-2 and KJD-3 were reinforced by the same measures, and two layers of CFRP were pasted at the column foot and joints in order to avoid premature failure of the plastic hinge area of the specimen. The column and cover beam were reinforced by enveloped steel, and the enveloped steel at the column foot was fixed on the base by a chemical anchor. The reinforcement design of specimens and on-site photographs are illustrated in Figure 5 and Figure 6, respectively. The enveloped steel ratio of column and cover beam is 2.33% and 3.11%, respectively, while the batten plate stirrup ratio is 0.77% and 0.79%, respectively, and the volume ratio of CFRP circumferential girth is 0.58%. As the first reinforcement step, the polymer cement mortar was used to repair the defective part after removing the surface of the loose concrete, and the second step was to inject mucilage to the cracks. Finally, CFRP and enveloped steel reinforcement construction were carried out after polymer cement mortar and mucilage solidification.

### 2.4. Measurements

**Figure 7:**
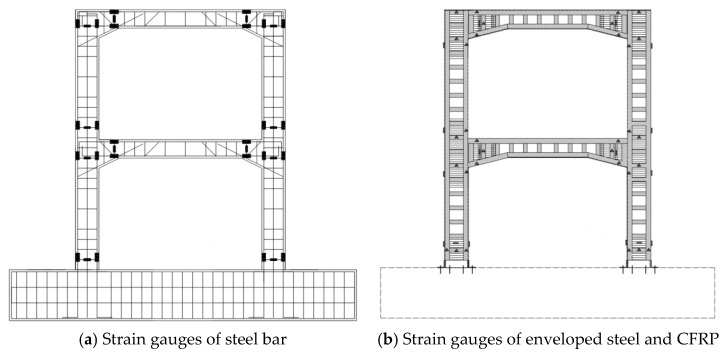
Layout of strain gauge.

The horizontal load and horizontal displacement were recorded by the actuator. The strain of longitudinal reinforcement and stirrup in the potential plastic hinge area of column, the strain of section steel and batten as well as the strain of the CFRP at the cover beam and column were measured and recorded by strain gauges. The obtained layout of the strains is illustrated in Figure 7.

## 3. Test Phenomenon

### 3.1. Specimen KJD-0

**Figure 8:**
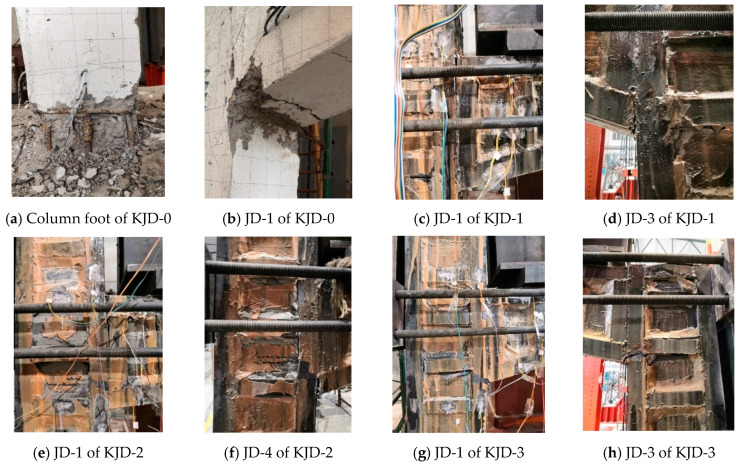
Typical failure phenomenon of specimens.

When the horizontal displacement reached 12 mm in the first cycle, micro-cracks appeared at the foot of the column marked as LZ1 and LZ3. The original cracks extended and penetrated, while new cracks appeared in the first cycle of 16 mm. The strain monitoring system indicated that the strain of the longitudinal bars at the foot of LZ1 and LZ3 columns exceeded the yield strain. As the loading continued, the concrete cover of column foot began to fall off, the longitudinal reinforcement basically yielded, and two fine cracks appeared at the bottom of beam GL1. When the horizontal displacement reached 63 mm, the horizontal bearing capacity reached the peak load, the concrete cover of the column foot spalled off seriously, and many longitudinal bars were exposed. At a horizontal displacement of 90 mm, the bearing capacity decreased to less than 85% of the peak load, and the test stopped. Figure 8a,b illustrate the failure mode.

### 3.2. Specimen KJD-1

At a horizontal displacement of ±18 mm, the strains of longitudinal bars, enveloped steel and splice plates at the foot of column LZ1 exceeded the yield strain. At a horizontal displacement of ±54 mm, the weld splice plate at the top of column LZ3 was pulled off. At a horizontal displacement of ±72 mm, the weld of enveloped steel of joint JD1 was pulled off. At the first cycle of ±99 mm, the enveloped steel on the top of column LZ4 completely fractured, and the CFRP of joint JD3 bulged slightly (Figure 8d). With the displacement reverse loading, the bulge partly recovered, and the horizontal bearing capacity reached the peak load. At ±117 mm, the CFRP of joint JD1 and JD3 bulged seriously, the core concrete was crushed, and the bearing capacity was significantly decreased.

### 3.3. Specimen KJD-2

At a horizontal displacement of ±18 mm, the strain of angle steel, steel plate of column foot of LZ1 exceed the yield strain. At the second cycle of ±54 mm, the splice plate on the top of column LZ3 bulged, and the weld connection of angle steel of joint JD1 cracked. At ±99 mm, the angle steel at the top column LZ2 fractured seriously, the original cracks continued to expand to fracture, and the bearing capacity reached the peak load. At the first cycle of ±117 mm, CFRP of joint JD3 bulged seriously, the angle steel and reinforcement plate of joint JD1 all fractured (Figure 8e), the CFRP of columns and cover beams fractured, and concrete was significantly crushed. At the first cycle of ±126 mm, the specimen was seriously deformed and the load dropped to below 85% of the peak load, at which the test was stopped.

### 3.4. Specimen KJD-3

At the first cycle of displacement of ±18 mm, the strain of angle steel at the foot of column LZ1 exceed the yield strain; the weld of enveloped steel of joint JD1 was pulled off, accompanied by cracking sounds. At 99 mm, the concrete of JD1 and JD3 was seriously crushed, CFRP was obviously bulged, the CFRP at the foot of column was fractured, and a small amount of concrete debris was spalled. Figure 8g,h illustrate the failure mode.

### 3.5. Test Phenomenon Comparison of Specimens

In the later failure stage of KJ-0, the concrete cover of the column foot spalled off seriously, and many longitudinal bars were exposed. Specimens KJD-1, KJD-2 and KJD-3 present similar failure mode, longitudinal bars and enveloped steel partially reach yield, but there was no obvious deformation or failure at the foot column. It can be seen that the CFRP and steel jackets can effectively prevent the concrete crushing. However, the enveloped steel fractured and CFRP bulged seriously at the joints. Compared with KJD-0, the ultimate displacement and the peak capacity of specimens KJD-1, KJD-2 and KJD-3 have significantly increased. The damage degree of reinforced specimens increases with the increasing pre-damage degree, and the damage of KJD-3 is largest.

## 4. Test Results Discussion

### 4.1. Hysteresis Curve

**Figure 9:**
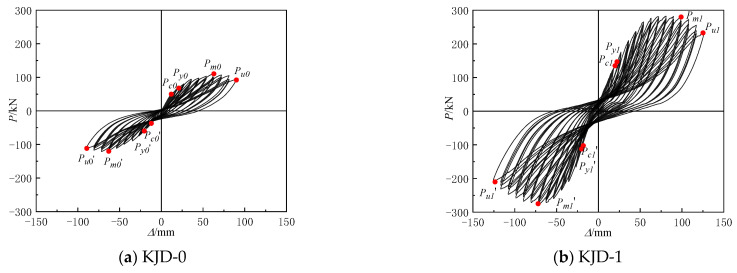

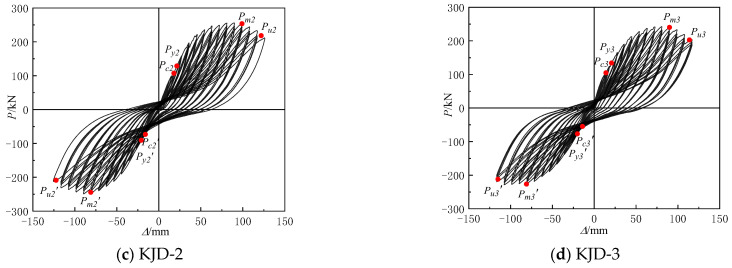
Hysteretic loops of specimens. (**a**–**d**). Note: For KJD-0, *P_c_* represents the cracking point of concrete; For KJD-1~KJD-3, *P_c_* represents the cracking point of surface glue; *P_y_*, *P_m_* and *P_u_* represent the yield point, peak point and limit point, respectively.

The recorded P-Δ curves of the four specimens are shown in Figure 9. At the beginning of the test, the lateral load is approximately in line with displacement. The hysteresis curve gradually tilts to the *X*-axis direction and exhibits an inverse s-shape, and the enclosed area gradually increased. In the yielding stage, the maximum load and hysteresis loop area are similar, and the degradation of stiffness and energy dissipation capacity is not significant under the same level of displacement loading, indicating that there is no obvious cumulative damage under the low circumferential reciprocal loading of the specimen. Compared with the prototype contrast specimen KJD-0, both the ultimate displacement and bearing capacity of the specimen KJD-3 have a great improvement, and the hysteresis loop increases significantly increased, which indicates that the seismic performance of the severely damaged specimen can still be effectively recovered after the composite reinforcement by CFRP and enveloped steel. The peak point and the slope of curve of the last two cycles are smaller than those of the first one, and the hysteresis loop area of the last two cycles also reduced to different degrees, indicating that the stiffness and energy dissipation capacity of the specimens are degraded. The degradations of KJD-2 and KJD-3 are greater than that of KJD-1, reflecting the effect of accumulated damage of the specimen under the low circumferential reciprocal loading.

There is no obvious inflection point on the skeleton curve of each specimen. The stiffness of each reinforced specimen is similar at the early stage of loading, and the initial stiffness of the comparison specimen is slightly lower. The reinforcement method of CFRP and enveloped steel exhibits a superior reinforcement effect in the early stage.

**Figure 10:**
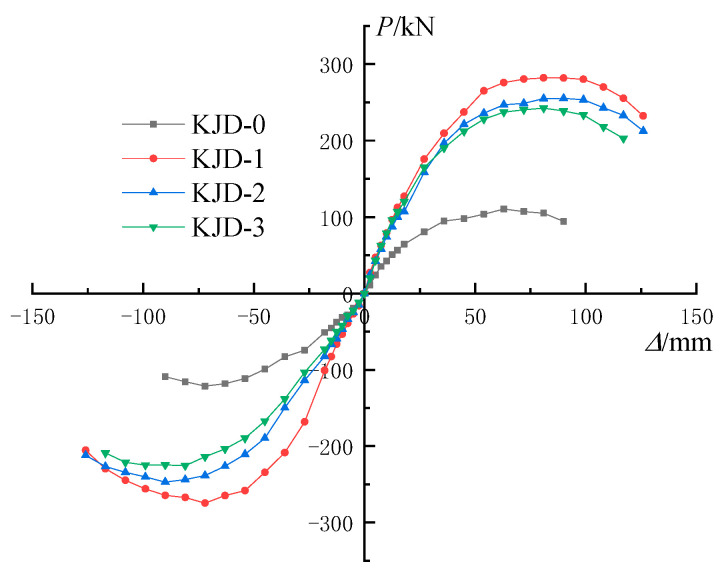
Skeleton curves of each specimen.

As is can be seen from Figure 10, compared with KJD-0, the ultimate displacement of reinforced specimens KJD-1, KJD-2 and KJD-3 has significantly increased. After the bearing capacity reached the peak load, CFRP and enveloped steel can effectively restrain the development of concrete cracks. The descending section of the skeleton curve is relatively gentle and exhibits a good ductility. The bearing capacity of specimens decreases faster with the increasing pre-damage degree. The ranking of the bearing capacity of the reinforcement specimens during forward loading is KJD-1 > KJD-2 > KJD-3, and significantly larger than KJD-0.

### 4.2. Ductility and Bearing Capacity

**Figure 11:**
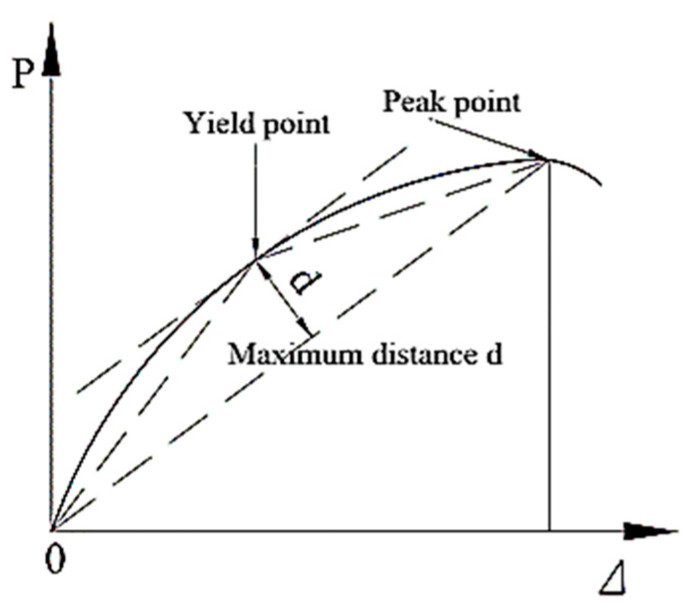
The farthest point method.

The displacement ductility coefficient is commonly used to express the plastic deformation performance of the specimen. Since there is no obvious yield point in the skeleton curve, the farthest point method proposed in the literature [21] is used to calculate the yield displacement, which is simple and feasible, and the calculation results are close to those of the graphical method and the equal energy method, and the schematic diagram of the farthest point method is shown in Figure 11. The displacement corresponding to the load dropping to 85% of the peak load is taken as the ultimate displacement. The displacement ductility factor μ is defined as the ratio of the ultimate displacement Δ*u* to the yield displacement Δ*y* [22]; it is calculated as follows:

(1) $$\mu =\frac{\Delta_{u}}{\Delta_{y}}$$

The displacement ductility coefficients of each specimen are shown in Table 4, in which *ημ* represents the increased value of displacement ductility coefficient. It is seen that the ductility coefficient of the reinforced specimen is significantly higher than that of the prototype contrast specimen, which indicates that the composite reinforcement of CFRP and enveloped steel can effectively improve the overall ductility of DVFP.

The peak load *P*_max_ and the ultimate displacement *u* of each specimen are shown in Table 5, and *ηP*_max_ and *ηu* are defined as the increased value of the bearing capacity and the ultimate displacement, respectively. Table 5 shows that the bearing capacity and ultimate displacement of the severely damaged specimens strengthened with CFRP and enveloped steel recover or even exceed the prototype contrast specimen KJD-0. By comparing the *P*_max_ and *u* of the reinforced specimens KJD-1, KJD-2, KJD-3, it is seen that the reinforcement effect of the specimens is related to the degree of damage. To be specific, the smaller the degree of damage is, the more significant the reinforcement effect is.

### 4.3. Strength and Stiffness Deterioration

The strength degradation coefficient *λ* is expressed as

(2) $$\lambda =P_{i,3}/P_{i,1}$$

**Figure 12:**
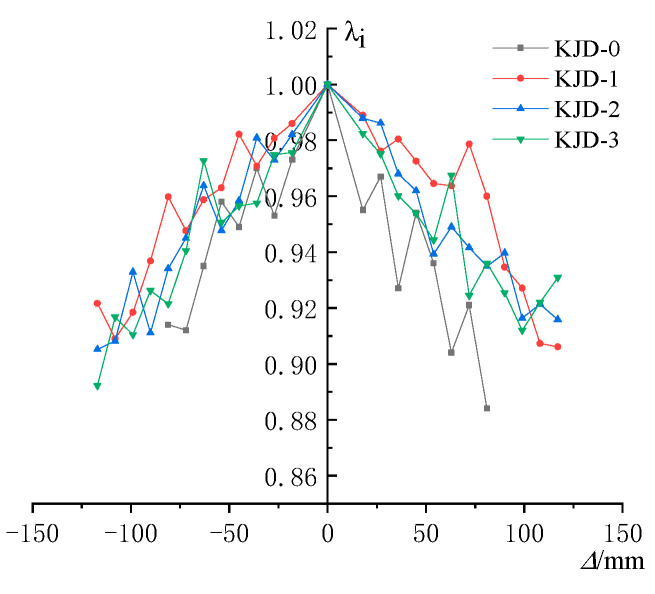
Strength degradation curves.

*P_i_*_,1_ and *P_i_*_,3_ represent the peak load of the first cycle and the third cycle at the *i*th drift level, respectively [23]. The relationships between *λ* and Δ are plotted in Figure 12. The stiffness degradation coefficient *K_i_* can be defined as

(3) $$K_{i}=\frac{\left|P_{m,i}^{+}\right|+\left|P_{m,i}^{-}\right|}{\left|\Delta_{m,i}^{+}\right|+\left|\Delta_{m,i}^{-}\right|}$$

**Figure 13:**
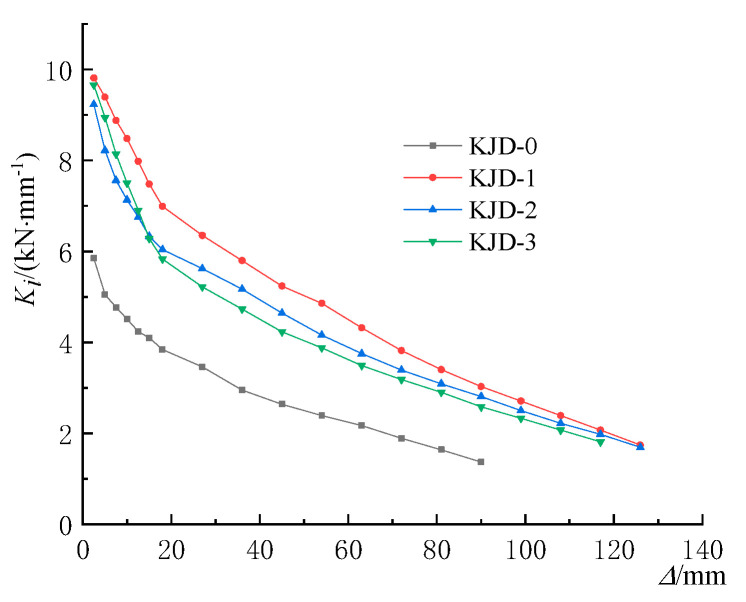
Stiffness degradation curves.

*P_m_*_,_*_i_* represents the peak load of the *i*th drift level in the half cycle; Δ*_m_*_,*i*_ represents the peak displacement of the *i*th drift level in the half cycle level [24]. The relationships between *K* and Δ are plotted in Figure 13.

As is can be seen from Figure 12, the strength degradation of the specimen after yielding is accelerated significantly due to the continuous damage accumulation. The strength degradation of the specimen KJD-0 was the most significant, and the ranking of the strength degradation of the reinforcement specimens during forward loading was KJD-3 > KJD-2 > KJD-1, which shows that the strength degradation is related to the degree of damage. The more serious the damage is, the faster the strength degradation is. The strength degradation coefficients are above 0.9, indicating that the reinforcement method is effective. The specimens maintain a more stable bearing capacity under large deformation and show good seismic performance.

The stiffness degradation curves are shown in Figure 13, and it is seen that the stiffness degradation coefficient *λ* of KJD-2 and KJD-3 is greater than that of KJD-0, but slightly lower than that of KJD-1; The stiffness degradation trends of all specimens are similar. At the beginning of the test, the bearing capacity and secant stiffness decreased gradually. As the loading continued, the secant stiffness decreased slowly. The stiffness degradation rate of KJD-2 was close to that of KJD-1 at the late loading stage, and that of KJD-0 was faster than that of KJD-3. This indicates that the composite reinforcement of CFRP and outer clad steel can improve the stiffness of the specimen and delay its stiffness degradation after the pier enters the nonlinear phase.

### 4.4. Energy Dissipation

**Figure 14:**
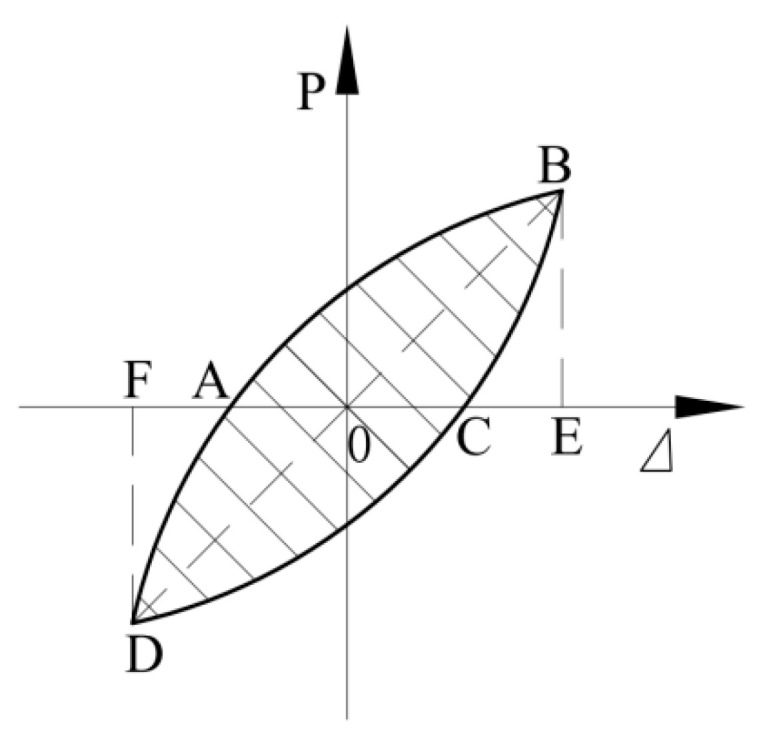
Energy dissipation index calculation.

**Figure 15:**
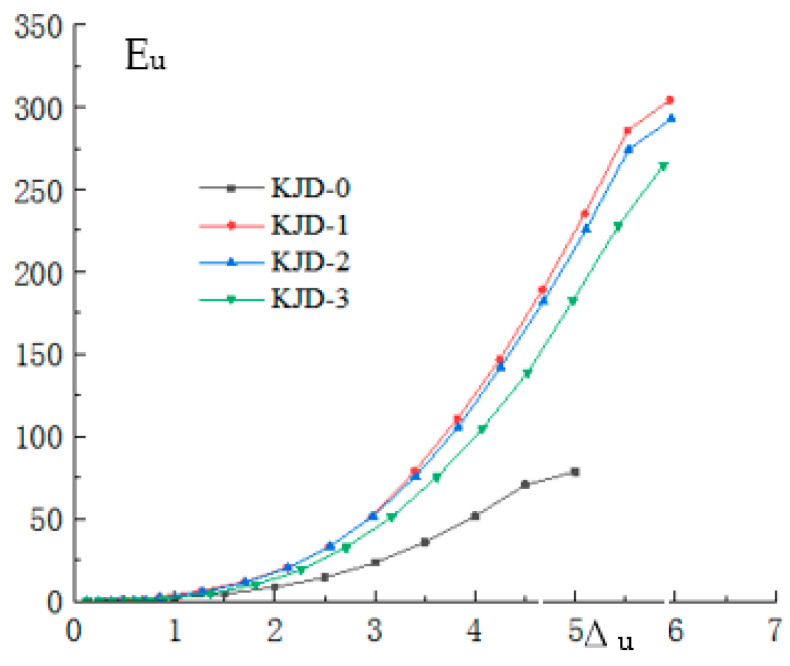
Cumulative energy dissipation curves.

The energy dissipation capacity of the specimens was evaluated using the cumulative hysteresis energy dissipation *E_u_* and the energy dissipation coefficient *E*. The calculation method of energy dissipation coefficient was shown in Figure 14 and its expression in Equation (4). The relationship between the cumulative hysteretic energy dissipation and the displacement ductility ratio *μ*∆ (the ratio of loading displacement to yield displacement) of each specimen is shown in Figure 15.

It is seen that: The varying trend of cumulative hysteretic energy consumption of each specimen is close to that of the displacement ductility ratio. In the elastic working stage, the displacement-ductility ratio is less than 1, and the accumulated hysteretic energy dissipation is close to 0. When the displacement ductility ratio is greater than 1, the cumulative hysteresis energy increases steadily with the increase in displacement ductility ratio, and the cumulative hysteresis energy of reinforcement specimens KJD-1, KJD-2 and KJD-3 is significantly higher than that of contrast specimen KJD-0 due to the participation of CFRP and outsourced steel. At the same level of displacement ductility ratio, the cumulative hysteresis energy of reinforcement specimens KJD-1, KJD-2 and KJD-3 decreases with the increase in pre-damage degree.

(4) $$E=\frac{S_{\left(ABC+CDA\right)}}{S_{\left(OBE+ODF\right)}}$$

*S_ABC_* is the area within the hysteretic loop; *S_OBE_* is the corresponding triangle area [25].

**Figure 16:**
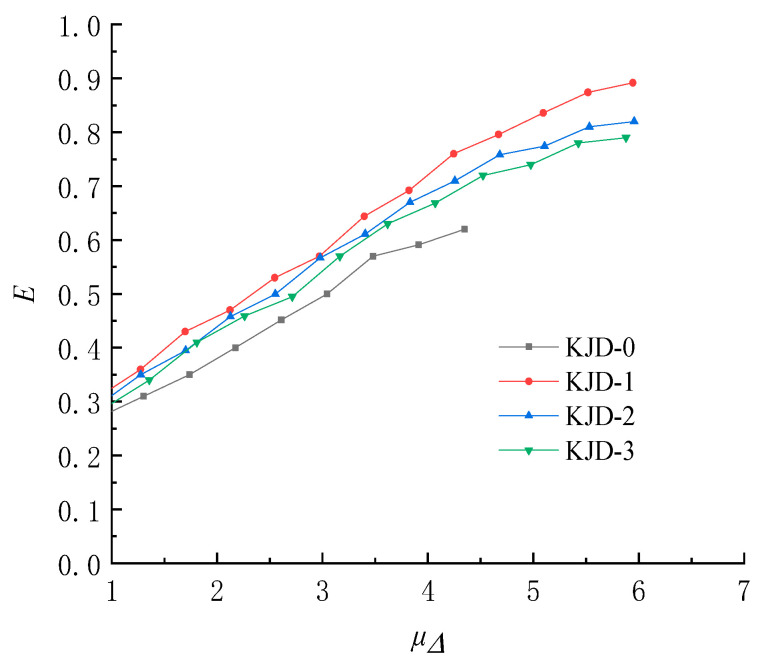
Energy dissipation coefficient curves.

The energy dissipation coefficient curve of each specimen is shown in Figure 16. When the displacement ductility coefficient is less than 1, the specimen is in the elastic working stage, and the coefficient is not significant to study. Therefore, only the energy dissipation coefficient after yielding is calculated. It is seen that, at yielding, the energy dissipation coefficients of all specimen are similar. With the increasing loading, *E* grows smoothly, and the growth rate of the comparison specimen KJD-0 is smaller than that of the reinforced specimens.

### 4.5. Residual Displacement Ratio

The unrecoverable permanent deformation of bridge pier after repeated loading is called residual displacement of bridge pier, and it is an important index for damage or repairability evaluation of bridge pier after earthquake. In this paper, the proposed residual displacement coefficient R is used to analyze the residual displacement variation of each specimen [26]. R is calculated according to the following formula:

(5) $$R=\frac{1}{2}\left(\frac{\Delta_{r1}}{\Delta_{y1}}+\frac{\Delta_{r2}}{\Delta_{y2}}\right)$$

∆*_r_*_1_ and ∆*_y_*_1_ represent residual deformation and yield displacement under positive loading, respectively, ∆*_r_*_2_ and ∆*_y_*_2_ represent residual deformation and yield displacement under negative loading, respectively.

**Figure 17:**
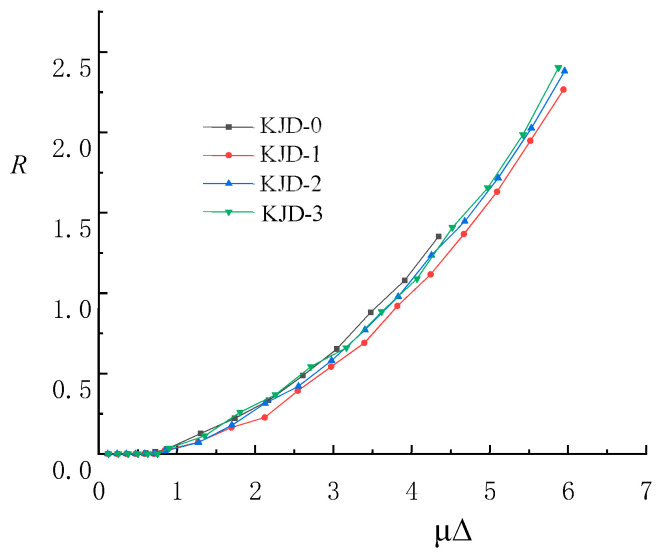
Residual displacement coefficient curves.

The residual displacement coefficient curve is shown in Figure 17. It is seen that the residual displacement coefficient of each specimen varies basically with the displacement ductility ratio. When the displacement ductility ratio is less than 1, the residual displacement coefficient is equal to 0. When the displacement ductility ratio is greater than 1, the residual displacement coefficient changes linearly with the displacement ductility ratio. Under the same displacement ductility ratio, the residual displacement coefficient of KJD-1 is lower than that of the prototype contrast specimen KJD-0, and the residual displacement coefficients of KJD-2 and KJD-3 are also slightly lower than that of the prototype contrast specimen KJD-0. It is shown that the composite reinforcement of CFRP and enveloped steel can slow down the growth of residual displacement coefficient.

## 5. Cumulative Damage

Low reversed cyclic loading results in structural damage, and the damage accumulates with the increase in the number of load cycles. When the damage reaches a threshold, part of the structure or the entire structure fails, and it cannot continue to bear the load. Several studies have proposed different seismic damage models, as shown in Table 6.

**Figure 18:**
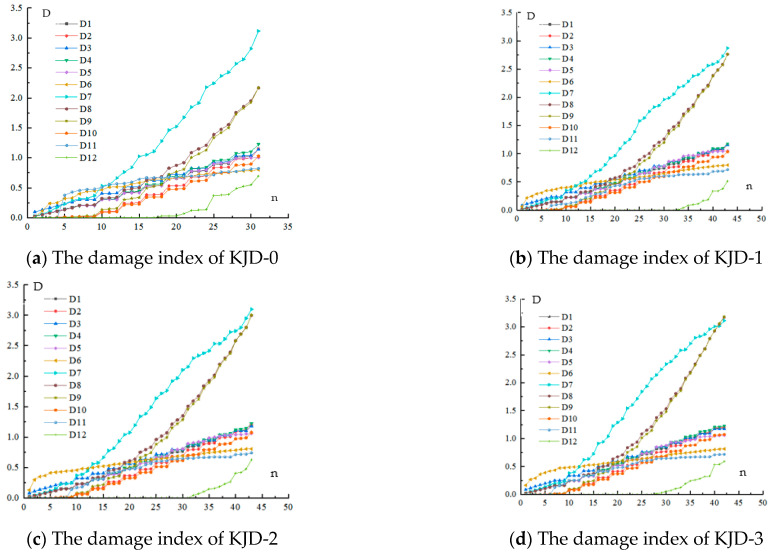
The damage index of KJD-0~KJD-3.

As shown in Figure 18, the damage index of the four specimens calculated with different damage models and the damage index development curve of each specimen was obtained. The analysis of the damage curves of four specimens shows that the damage index calculated by different models is more discrete under the same damage state, mainly because each damage model is based on limited parameters of different components or structural test data. Another reason may be that the structural design, test conditions, and loading methods will affect the calculation results.

As is shown in Figure 18, except for D6 [32], the development trends of damage indexes of all specimens calculated by different damage models are roughly similar, and the growth rate is slow in the early stage. With the progress of cyclic loading, the exponential growth accelerates after the specimen enters the yield stage, which can effectively reflect the cumulative damage of specimens during the loading process.

D6 increases rapidly in the early stage, but tends to be flat in the later stage, which is inconsistent with the actual damage process of the specimen. D2 [28] and D9~D12 [35,36,37,38] have less damage before yielding, which is consistent with the actual situation in the early stage. The damage index of D7~D9 [33,34,35] in the later stage exceeds 1, which overestimates the damage degree of the specimen during the loading process. When the specimen reaches moderate damage, the damage index of D12 [38] is still small, which does not conform to the actual situation. The damage index calculated by D5 [31], D10 [36], and D11 [37] is small in the early stage, and gradually increases during the loading process.

The damage phenomenon and the measured data of the specimen during the loading process can provide a lot of evidence for seismic-damage research of DVFP. Whether the earthquake damage model can better evaluate the damage degree of the specimen during the loading process mainly depends on the damage index calculated by the model and the test results. Objective and accurate damage evaluation criteria are the premise for quantitatively evaluating the damage degree of bridge piers. It is seen from Figure 18 that the Kumar model [36] can better reflect the damage evolution of DVFP during the test process, and the development trend of the calculation results is consistent with the actual damage situation.

## 6. Conclusions

(1) The damage patterns of reinforced specimens with different degrees of seismic damage are similar, a fracture failure of the splice plate weld appeared at the joint, and a tear damage appeared at column foot. The damage degree of the column was greater than that of the cover beam.

(2) CFRP and enveloped steel can effectively improve the bearing capacity of the seismic-damaged specimens. Compared with the prototype contrast specimen, the peak loads of the moderately damaged and severely damaged specimens after reinforcement increased by 120.74% and 105.36%, respectively; the reinforced specimens had undulations in the process of bearing capacity degradation and the bearing capacity degradation coefficients were above 0.9.

(3) After the specimens reached the yield load, the CFRP and enveloped steel could effectively restrain the development of concrete cracks and improve the ductility. Compared with the prototype contrast specimen, the ultimate displacements of the strengthened moderately damaged and strengthened severely damaged specimens increased by 35% and 25.98%, respectively; the displacement ductility coefficients increased by 32.33% and 31.41%, respectively. Energy dissipation coefficients and residual displacement coefficients of the four specimens showed the same trend with the increase in displacement ductility ratio. The stiffness degradations of all specimens were similar, with faster degradation at the beginning of loading and leveling off after yielding.

(4) Kumar model can better reflect the damage evolution of strengthened seismic-damaged DVFP during the test process according to the comparative analysis of the calculation results, and the development trend of the calculation results is consistent with the actual damage situation.

## Figures and Tables

**Table 1:** Material properties test results of steel.

Steel	$f_{y}/MPa$	$f_{u}/MPa$	$E_{s}/MPa$
A8	279.3	478.6	2.1 × 10^5^
C10	377.5	576.8	2.0 × 10^5^
C14	369.8	569.4	2.0 × 10^5^
3 mm	304.2	438.6	2.1 × 10^5^
5 mm	316.1	463.5	2.1 × 10^5^
∟50 × 5	295.3	475.4	2.1 × 10^5^

**Table 2:** Material properties of CFRP and mucilage.

Material	Tensile Strength/MPa	Elastic Modulus/MPa	Elongation/%
CFRP sheets	3425	2.32 × 10^5^	1.63
CFRP sheets mucilage	52.7	2.82 × 10^3^	1.71
Poured mucilage	50.3	3.06 × 10^3^	1.67

**Table 3:** Pre-damage and reinforcement method of specimens.

Specimen	Damage Degree	Corresponding Failure Phenomenon	Lateral Drift/mm	Strengthening Method
KJD-0	—	—	—	—
KJD-1	—	—	—	CFRP and enveloped steel
KJD-2	Moderate damage	cracks of column foot and joints appeared	36	CFRP and enveloped steel
KJD-3	Severe damage	the bearing capacity reached the peak load	63	CFRP and enveloped steel

**Table 4:** Displacement ductility factors of specimens.

Specime- ns	Δ*_y_*/mm			Δ*_u_*/mm			*μ*		*μ_AVE_*	*ημ*/%
	Forward	Reverse		Forward	Reverse		Forward	Reverse		
KJD-0	21	20.4		90	89.3		4.29	4.38	4.33	—
KJD-1	22.3	20.1		125.2	121.6		5.61	6.05	5.83	34.64
KJD-2	21.5	20.8		119.9	122.3		5.58	5.88	5.73	32.33
KJD-3	20.7	19.1		110.9	115.1		5.36	6.03	5.69	31.41

**Table 5:** Comparison between mean *P*_max_ and Δ_u_.

Specimens	$\bar{P}$_max_/kN	*η*$\bar{P}$_max_/%	$\bar{\Delta }$_u_/mm	*η*$\bar{\Delta }$_u_/%
KJD-0	113.8	—	89.65	—
KJD-1	278.3	144.55	123.4	37.65
KJD-2	251.2	120.74	121.1	35.08
KJD-3	233.7	105.36	113	26.05

**Table 6:** Damage model calculation method.

	Author	The Calculation Formula
D1	Park-Ang [27]	$D=\frac{\delta_{m}}{\delta_{u}}+\beta \frac{\int dE}{F_{y}\delta_{u}}$
D2	Kunnath [28]	$D=\frac{\delta_{m}-\delta_{y}}{\delta_{u}-\delta_{y}}+\beta \frac{\int dE}{F_{y}\delta_{u}}$
D3	Niu [29]	$D=\frac{\delta_{m}}{\delta_{u}}+\alpha {\left(\frac{E}{E_{u}}\right)}^{\beta_{1}}$
D4	Fu [30]	$D=\frac{\delta_{m}}{\delta_{u}}+\frac{\sum e_{i}E_{i}}{F_{y}\delta_{u}}$ $e_{i}=\frac{1}{\delta_{im}/\delta_{y}}\log_{\frac{\delta_{u}}{\delta_{y}}}\left(\frac{\delta_{im}}{\delta_{y}}\right)$
D5	Luo [31]	$D=\frac{\delta_{m}}{\delta_{u}}+\left(1-\frac{\delta_{m}}{\delta_{u}}\right)\frac{\beta \sum_{i}{\left(\delta_{m,i}^{\pm }/\delta_{u}\right)}^{\gamma }E_{i}}{F_{y}\delta_{u}}$
D6	Diao [32]	$D=\frac{K_{0}\Delta_{i}^{2}-\left(\int_{0}^{\Delta_{i}}f_{1}\left(\Delta_{i}\right)d\Delta_{i}+\int_{\Delta_{p}{}_{i}}^{-\Delta_{i}}f_{2}\left(-\Delta_{i}\right)d\Delta_{i}\right)}{K_{0}\Delta_{i}^{2}}$
D7	Fu [33]	$D=e^{\left(0.13u_{m}-0.39\right)}\frac{\delta_{m}}{\delta_{u}}+e^{\left(3.35-0.8u_{m}\right)}\frac{\beta \int dE}{F_{y}\delta_{u}}$
D8	Chen [34]	$D=(1-\beta )\frac{\delta_{m}}{\delta_{u}}+\beta \frac{E_{i}}{F_{y}(\delta_{u}-\delta_{y})}$
D9	Lu [35]	$D=(1-\beta )\frac{\delta_{m}-\delta_{y}}{\delta_{u}-\delta_{y}}+\beta \frac{E_{i}}{F_{y}(\delta_{u}-\delta_{y})}$
D10	Kumar [36]	$D=(1-\beta )\sum_{1}^{n_{i}}{\left(\frac{\delta_{m}-\delta_{y}}{\delta_{u}-\delta_{y}}\right)}^{c}+\beta {\sum_{1}^{n_{j}}\left(\frac{E_{j}}{F_{y}(\delta_{u}-\delta_{y})}\right)}^{c}$
D11	Yu [37]	$D=\left(1-\frac{k_{i}}{k_{0}}\right)+\alpha \left(\frac{k_{i}}{k_{0}}\right){\left(\frac{\sum \delta_{pi}}{\delta_{y}}\right)}^{\beta }$
D12	Chen [38]	$D=\left(1-\frac{k_{i}}{k_{0}}\right)+\frac{\beta \sum {\left(\delta_{m}/\delta_{u}\right)}^{\gamma }E_{i}}{F_{y}\delta_{u}}\frac{k_{i}}{k_{0}}$

Note: *δ_m_* is the maximum deformation, *δ_n_* is the ultimate deformation under monotonic loading, *δ_y_* is the yielding deformation, *Q_y_* is the yielding strength, *E_i_* represents the hysteretic energy by one element, *β_i_* is a weighting factor of the energy term, where *K*_0_ is the initial stiffness of the structure and represents the top displacement of the structure under cyclic positive and negative peak loads.

## Data Availability

Not applicable.
